# Detecting and characterizing special nuclear material for nuclear nonproliferation applications

**DOI:** 10.1038/s41598-023-36171-8

**Published:** 2023-06-27

**Authors:** S. A. Pozzi, Z. He, J. Hutchinson, I. Jovanovic, R. Lopez, K. Ogren, J. Nattress, D. Shy, S. D. Clarke

**Affiliations:** 1 Department of Nuclear Engineering and Radiological Sciences, University of Michigan, Ann Arbor, MI 48109 USA; 2 Oak Ridge National Laboratory, Oak Ridge, TN 37830 USA; 3 Los Alamos National Laboratory, Los Alamos, NM 87545 USA

**Keywords:** Physics, Engineering, Electrical and electronic engineering

## Abstract

There is an urgent need for new, better instrumentation and techniques for detecting and characterizing special nuclear material (SNM), i.e., highly enriched uranium and plutonium. The development of improved instruments and techniques requires experiments performed with the SNM itself, which is of limited availability. This paper describes the findings of experiments performed at the National Criticality Experiments Research Center conducted using new instruments and techniques on unclassified, kg-quantity SNM objects. These experiments, performed in the framework of the Department of Energy, National Nuclear Security Administration Consortium for Monitoring, Technology, and Verification, focused on detecting, characterizing, and localizing SNM samples with masses ranging from 3.3 to 13.8 kg, including plutonium and highly enriched uranium using prototype detectors and techniques. The work demonstrates SNM detection and characterization using recently-developed prototype detection systems. Specifically, we present new results in passive detection and imaging of plutonium and uranium objects using gamma-ray and dual particle (fast neutron and gamma-ray) imaging. We also present a new analysis of the delayed neutron emissions during active interrogation of uranium using a neutron generator.

## Introduction

The safety and security of nuclear weapons in nuclear weapons states and the prevention of acquisition of nuclear weapons by non-nuclear weapons states are great challenges that we face in society today. Nuclear nonproliferation, safeguards, and arms control agreements and treaties help counter the growing threat of nuclear weapons. New technologies are needed for the timely detection of any activity not in compliance with current nuclear treaty obligations.

Nuclear weapons rely on kg-quantities of special nuclear material (SNM, primarily highly enriched uranium and plutonium) for the fissile explosive. Hence, detecting and characterizing the SNM is a major research focus whose technical outcomes have the potential to strengthen existing and new agreements and policies. Current instrumentation includes gamma-ray imaging and spectroscopy^1–3^, neutron-scatter imaging^4,5^, and active interrogation^6^. The development of new, better instrumentation and techniques for the detection and characterization of SNM requires experiments performed with the material itself. SNM experiments are necessary to test the radiation detection systems and the results are used to improve their design and performance. The availability of SNM is very limited, and often the release of results from experiments with SNM is restricted.

### Prior work

Experiments with sub-critical, near-critical, and critical SNM configurations were conducted in previous work. The Planet vertical lift machine at Los Alamos National Laboratory was used to assemble highly enriched uranium in configurations from subcritical up to critical. Neutron and gamma-ray emissions were measured using an array of organic scintillation detectors to characterize the neutron multiplication of each configuration^7,8^. For subcritical configurations of nuclear material, where the fission chain reaction is not fully self-sustaining, the multiplication is quantified using the subcritical multiplication factor (M). This factor, which quantifies the increased neutron emission rate due nuclear fission, is 1.0 for non-multiplying configurations and increases as the configuration approaches critical (self-sustaining fission reaction). Subcritical masses of plutonium have also been characterized using organic scintillation detectors; in these experiments, the plutonium was surrounded by various reflectors to produce a range of neutron multiplication^9^. Initial neutron and gamma-ray imaging experiments with uranium and plutonium have been performed using a dual-particle imaging system^10^; for the uranium experiments, a neutron generator was used to induce fission^11^. Experiments using CdZnTe imaging systems to detect and characterize SNM demonstrated the use of the systems to estimate plutonium grade^12^.

### Present research

The work discussed here was conducted on unclassified, kg-quantity SNM objects in the framework of the Department of Energy, National Nuclear Security Administration university consortia. The work includes multiple experiments performed with prototype detectors and algorithms that demonstrate SNM detection and characterization beyond the state of the art. Specifically, we present new results in passive detection and imaging of plutonium and uranium objects using CdZnTe imaging^13^ and dual particle imaging^14^. This work presents novel results on imaging large volumes of SNM in an extended geometry using the CdZnTe-TEI combination. In our dual particle imaging results, we show the first gamma ray image measured with a stilbene/CeBr system gated on gamma ray energies of interest to nonproliferation applications (i.e., the Pu-239 emission energies). For the characterization of shielded uranium, one needs to use active interrogation to induce signatures to characterize the SNM. For completeness, we present results demonstrating shielded uranium characterization. The technique uses delayed neutron emissions during active interrogation of uranium using a neutron generator^15–17^.

## National Criticality Experiments Research Center

### Facility

The National Criticality Experiments Research Center (NCERC) was founded in 2011 and is located inside the Device Assembly Facility (DAF) at the Nevada National Security Site (NNSS)^12^. NCERC is operated by Los Alamos National Laboratory (LANL) and contains assemblies and material that were previously used at the Los Alamos Critical Experiments Facility (LACEF) from 1946 to 2006^18^. NCERC is currently the only general-purpose critical experiments facility in the US and one of only a few that remain operational throughout the world.

NCERC has a very large amount of SNM and four critical assembly machines available for experiments^19–22^. The first focus area at NCERC is related to criticality experiments that explore reactivity phenomena and are primarily used to support improved nuclear data for DOE/NNSA missions. The second focus area is hands-on activities with static subcritical assemblies for radiation detector testing. For both focus areas, the projects include both new experimental activities as well as training courses (in the areas of criticality safety, reactor safety, and radiation detection.

Since 2015, experiments have been performed at NCERC in support of the Defense Nuclear Nonproliferation (DNN) university consortia: five experimental campaigns from 2015 to 2019 in support of the Consortium for Verification Technology and Consortium for Nonproliferation Enabling Capabilities, and one experimental campaign in 2021 in support of the Consortium for Monitoring, Technology, and Verification, and the Consortium for Enabling Technologies and Innovation. NCERC helps the missions of these consortia by providing large quantities of nuclear material used to test new technologies.

### Special nuclear material objects

The majority of the experiments that have been performed at NCERC for the DNN university consortia have involved spherical metal systems with large quantities of SNM. For some systems (in particular imaging systems), multiple SNM items were often used in the experiment at the same time.

**Figure 1:**
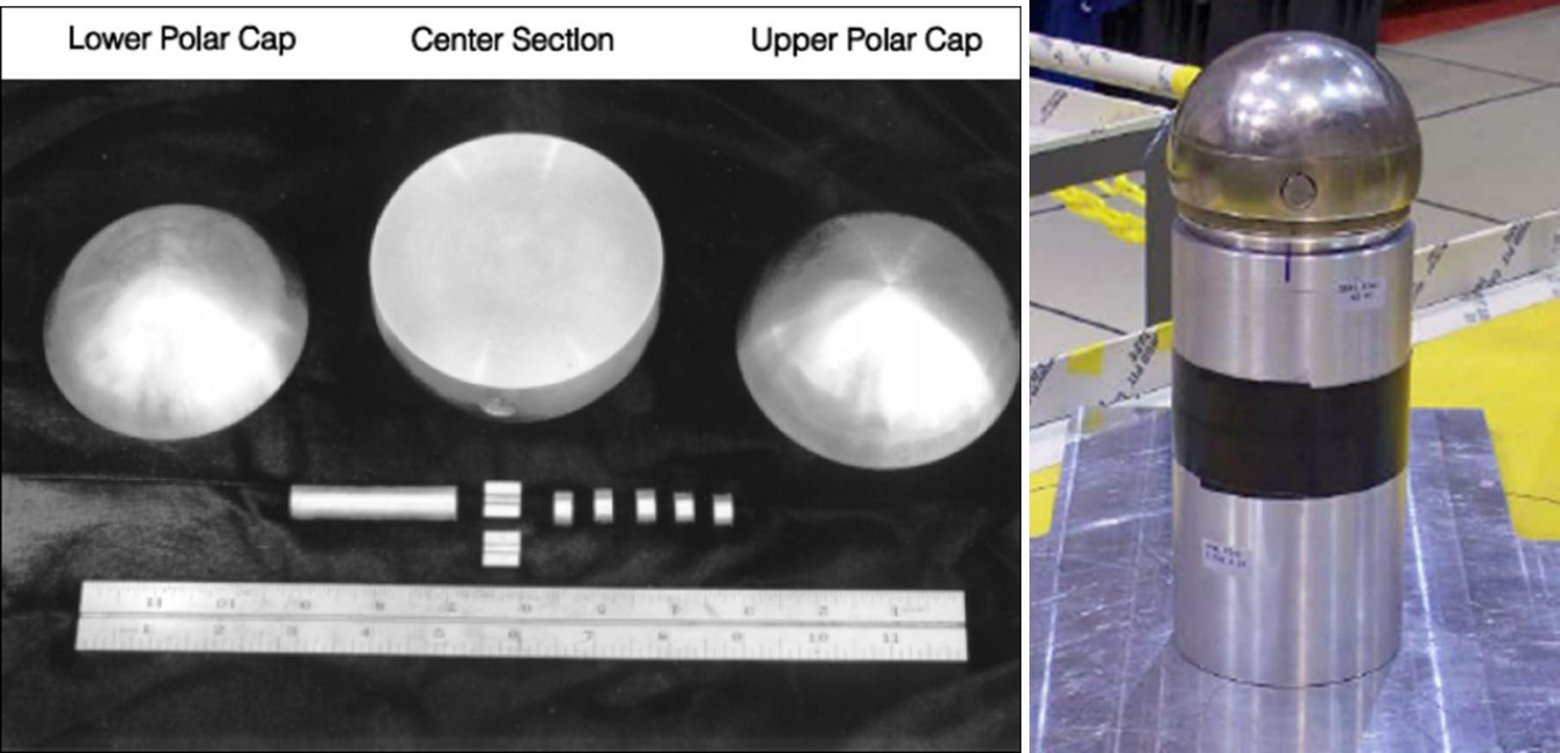
Thor core components and fully assembled spherical assembly.

The Thor core consists of three pieces of weapons-grade delta-phase Pu that have a combined mass of approximately 9.5 kg^23^. The three pieces, when combined, form a roughly spherical shape. Various combinations of the three pieces (along with 1.27-cm diameter Pu cylinders that can be inserted in the center piece) can be used to achieve a wide range of Pu masses, resulting in a large range of system multiplication and neutron leakage, which is the amount of neutrons escaping the sample. Figure 1 shows a photo of the Thor core components as well as the fully assembled spherical assembly. The masses of the three major components are 3273.9 g for the upper section, 4158.2 g for the center section, and 2216.9 g for the lower section^23^.

**Figure 2:**
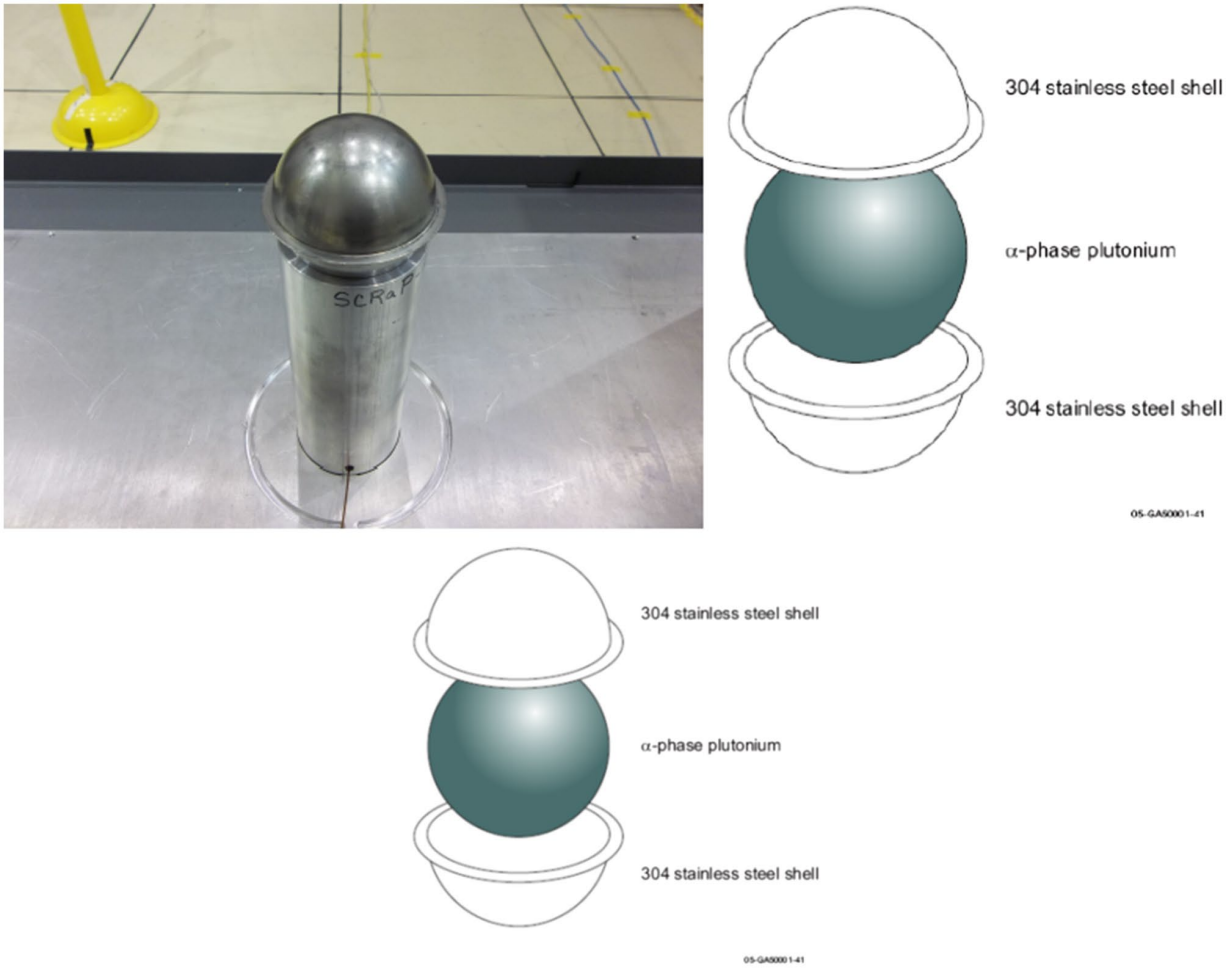
Photograph and schematic of the beryllium reflected plutonium (BeRP) ball.

The beryllium reflected plutonium (BeRP) ball is a sphere of weapons-grade alpha-phase Pu with a mass of approximately 4.5 kg^24^. It was cast and clad in stainless steel in 1980 for use in a Planet experiment in which it was reflected by beryllium (Be)^25^. Figure 2 shows a photograph of the BeRP ball. The BeRP ball is a very interesting object for detector geometry testing because it has a high subcritical multiplication factor (near 4.5) and large neutron output (near 10^6^ neutrons per second)^24^. The BeRP ball has been used in many experiments throughout the years, many of which include adding reflectors around the sphere of different materials including polyethylene, lucite, nickel, and copper^26–29^. Measurements with the BeRP ball are often used for nuclear data and/or analytical methods validation^30^.

**Figure 3:**
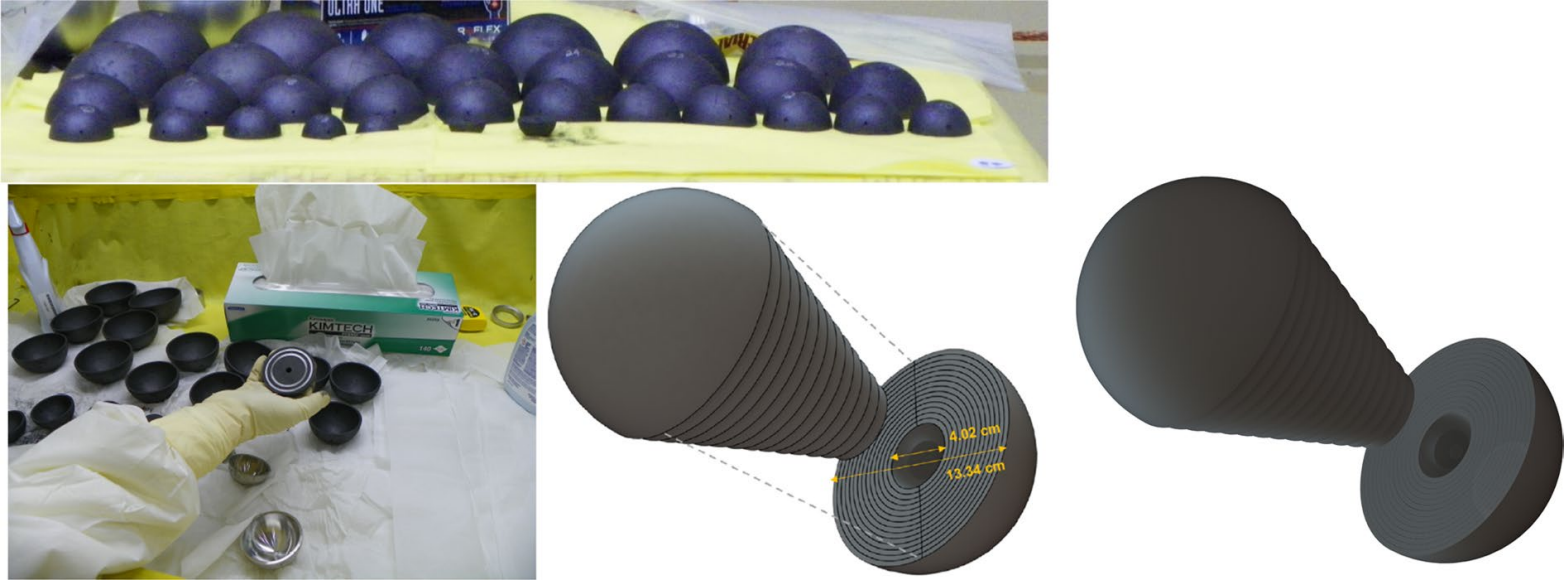
Photograph and schematic of the Rocky Flats shells. The inner and outer diameters of the assemblies are 4.02 and 13.34 cm, respectively.

The Rocky Flats (RF) shells are nesting 0.3175-cm-thick hemishells of HEU metal^31,32^. There are 80 hemi-shells total, resulting in well over a critical mass of bare HEU. Figure 3 shows a photograph of the RF shells. These shells are convenient for testing detector technology because one can build different combinations of shells, allowing for varying mass and geometry.

**Figure 4:**
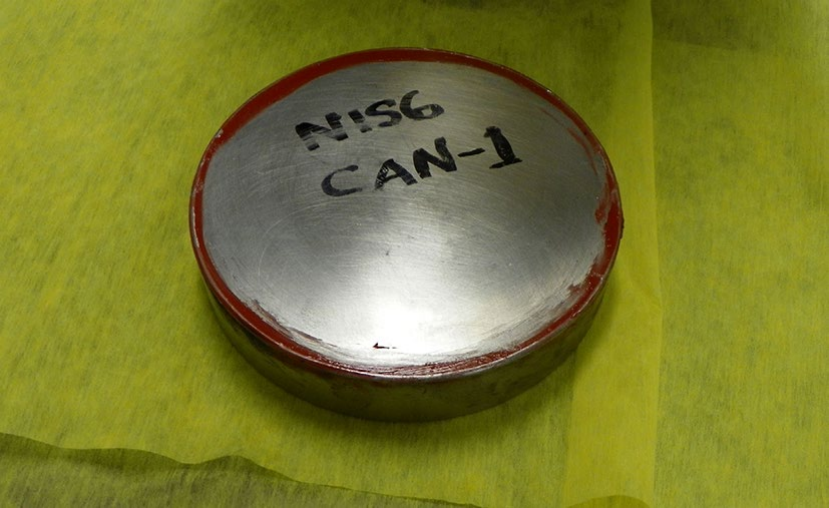
Photograph of the NIS6-CAN-1 Sintered PuO_2_.

The NIS6-CAN-1 is a sintered PuO_2_ sample (diameter of 15.24 cm and height of 4 cm). A photograph of this sample is shown in Fig. 4. This sample contains approximately 3.3 kg of Pu with 6% ^240^Pu. This item is of interest in that it emits neutrons the ^18^O(α,n)^21^Ne and ^17^O(α,n)^20^Ne reactions in addition to spontaneous fission in the ^240^Pu. Due to its geometry, this sample has little multiplication.

## Experimental setups

### CdZnTe gamma-ray imaging

This section presents gamma-ray imaging results of the THOR core center section and BeRP ball with a gamma-ray imaging system based on CdZnTe. The setup includes a detector and a time-encoding system. The detector is composed of a 3 × 3 array of pixelated CdZnTe, each with a volume of 2 × 2 × 1.5 cm^3^. The high resolution detector has an energy resolution of better than 0.35% full-width-at-half-maximum at 662 keV for single pixel events^33^. The time-encoding system consisted of a rank 79 MURA coded aperture with a pixel pitch of 1.4 mm mounted on a translational stage that can move the mask horizontally and vertically relative to the detector. A detailed description of the time-encoded system is available in^34^.

**Figure 5:**
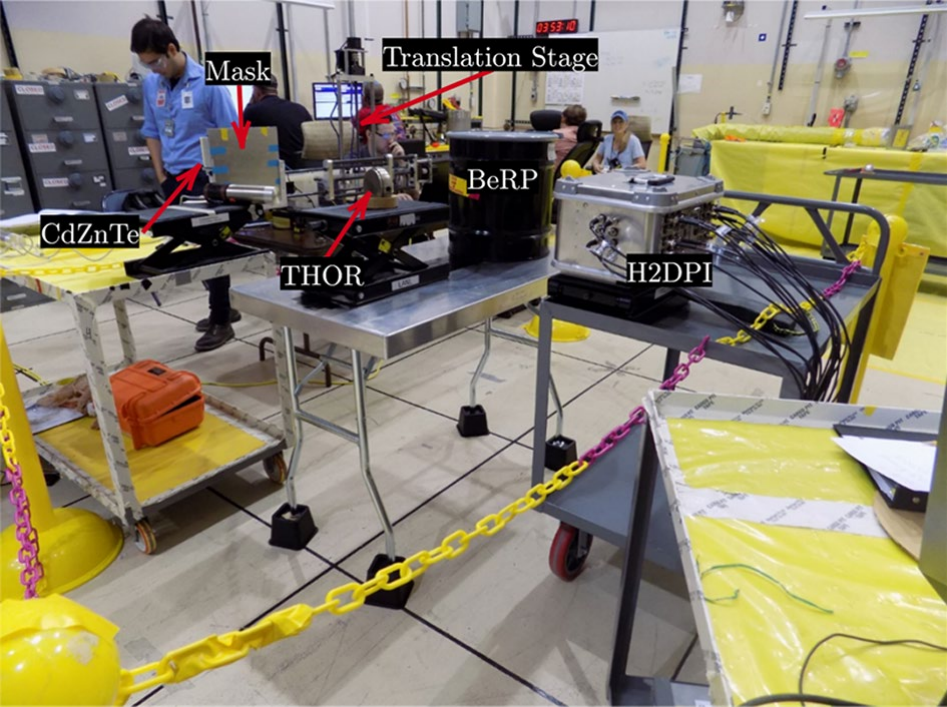
Experimental setup for gamma-ray imaging of the THOR core and BeRP ball plutonium objects.

The plutonium objects were both placed 100 cm from the plane of the coded mask, with the THOR core center section placed on its side so that the edge of the cylinder is facing the imaging system. To maintain a large field of view, the mask to detector distance was kept at 9 cm which configures the system into a low-magnification coded aperture mode. Figure 5 shows the experimental setup. Additionally, the center section of the Thor core diameter is hollow, as shown in Fig. 1, presenting an interesting challenge to image and resolve. The hole was aligned by eye such that it is inline radially from the CdZnTe imager.

### Dual-particle imaging

**Figure 6:**
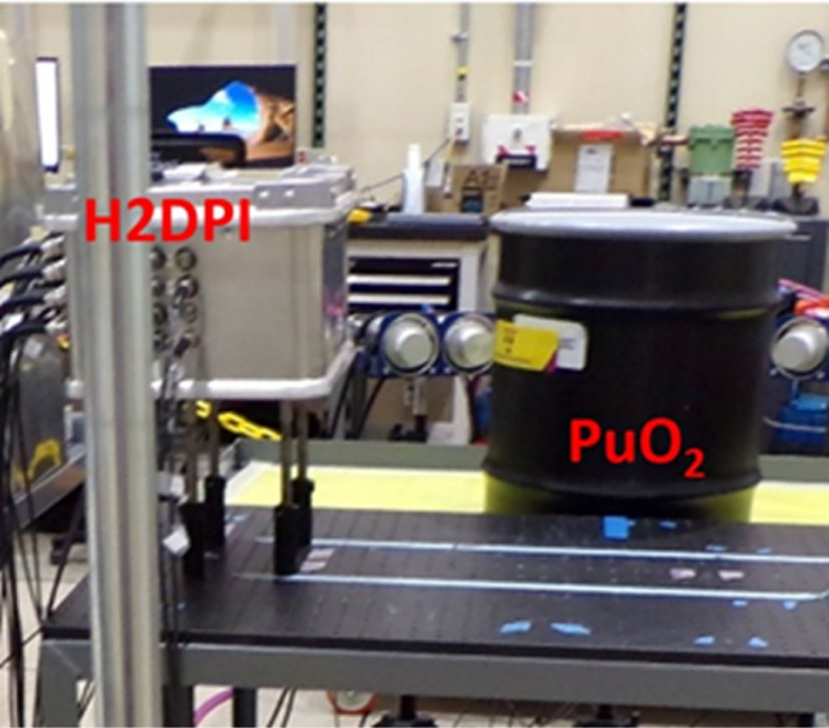
Photograph of the experimental setup showing the H2DPI and the plutonium oxide storage barrel.

SNM emits both neutrons and gamma rays. A handheld dual-particle imaging system (H2DPI) was developed to detect both types of particles^14^. An imaging experiment was performed using the NIS6-CAN-1 sintered PuO_2_ source with a neutron emission rate of approximately 400,000 neutrons per second. According to a radiograph taken at NCERC, the sample was situated 0.4 cm from the bottom of the inner storage container. The sample was placed in a specific (azimuthal, altitude) angular direction with respect to the front of the imager in order to compare position reconstruction after the measurement. The PuO_2_ was placed 63.4 cm away at (− 51.96°, 25.26°). Figure 6 shows a photograph of the experimental setup.

The H2DPI consists of twelve 6 × 6 × 50 mm^3^ stilbene bars for imaging gamma rays and fast neutrons and eight 6 mm height, 6 mm diameter CeBr_3_ inorganic scintillator cylinders to improve gamma ray detection efficiency. The radiation detection components are coupled on top and bottom ends to two silicon photomultiplier arrays that are used for the readout. The imager itself as well as sources are mounted on an optical breadboard to ensure accurate placement and orientation. The device is a passive imaging system that is capable of imaging in a complete 360° field of view.

### Active neutron interrogation

These experiments focus on neutron active interrogation techniques for characterizing uranium objects based on measurement of induced delayed-neutron signatures. We demonstrate uranium isotopic discrimination^15^ and estimation of enrichment based on measurement of the buildup and decay time profiles of long-lived delayed neutron groups^16,17^. These profile shapes can be derived from nuclear data and provide a method for estimating uranium enrichment without the need for a calibration standard. Additionally, we examine the effects of re-interrogation by delayed neutrons on the overall delayed signal. These effects are strongly dependent on the cross-section for fission induced by delayed neutrons, and can thus provide an additional means of discriminating uranium isotopes. Finally, we investigate the effects of neutron-moderating shielding on delayed neutron time profile measurements and find that delayed neutron profiles may still provide a robust discrimination signature in certain shielding scenarios.

The primary experimental test objects were the Rocky Flats shells. In most of the experimental configurations, the shells were arranged to form a solid sphere with a mass of approximately 13.8 kg. A set of concentric depleted uranium (DU) shells with similar mass (12.8 kg) was also used as a test object to provide a point of comparison between ^235^ and ^238^U induced delayed neutron signatures. In each experiment, the HEU and DU objects were interrogated with 14.1-MeV neutrons from a pulsed DT generator, which was operated in a series of on/off cycles. The DT generator was operated at 100 Hz with a pulse width of approximately 10 microseconds, resulting in a nominal 4-pi flux of approximately 10^8^ n/s. In each on/off cycle, the generator was turned on for 30–60 s (depending on the experiment) and off for 60 s. The buildup of delayed neutrons was recorded between pulses while the generator was on, and the decay of the delayed neutron population was recorded during each time period that the generator was turned off.

**Figure 7:**
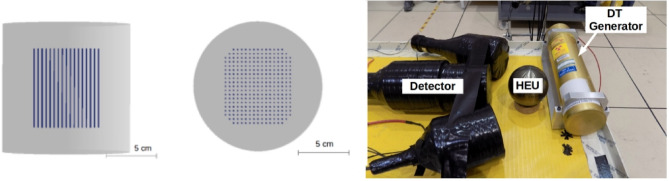
(left) Side and (middle) top model view of the detector and (right) the experimental setup for one of the measurement campaigns showing the neutron source and object (Adapted from^15^).

A custom heterogeneous composite detector was used to detect neutrons from neutron-induced fission. The detector has a height and diameter of 12.7 cm^35^. An array of enriched Li-6 scintillating glass (GS20) square rods (1 × 1 × 7.6 cm^3^) was embedded and centered in a scintillating polyvinyl toluene (PVT) matrix. Neutrons interacting with the detector typically undergo thermalization in the PVT and are later captured in the lithium-doped glass. The neutron capture signal has a distinct light output corresponding to the Q-value (4.8 MeV) of the reaction. Neutron-capture events are further distinguished from gamma-ray and neutron elastic-scatter events based on their pulse shape, which is governed by the scintillation properties of the glass. The glass and PVT matrix have different scintillation decay constants, easily achieving accurate particle identification with simple charge integration techniques. Neutron elastic scatters are identified via a time gate preceding a neutron capture. The composite detector essentially has no energy threshold for neutron detection, increasing its sensitivity to low-energy (on the order of hundreds of keV) delayed neutrons. Figure 7 shows side and top views of the detector and the detector in one of the experimental configurations, along with the neutron source and object.

## Results

### CdZnTe gamma-ray imaging

**Figure 8:**
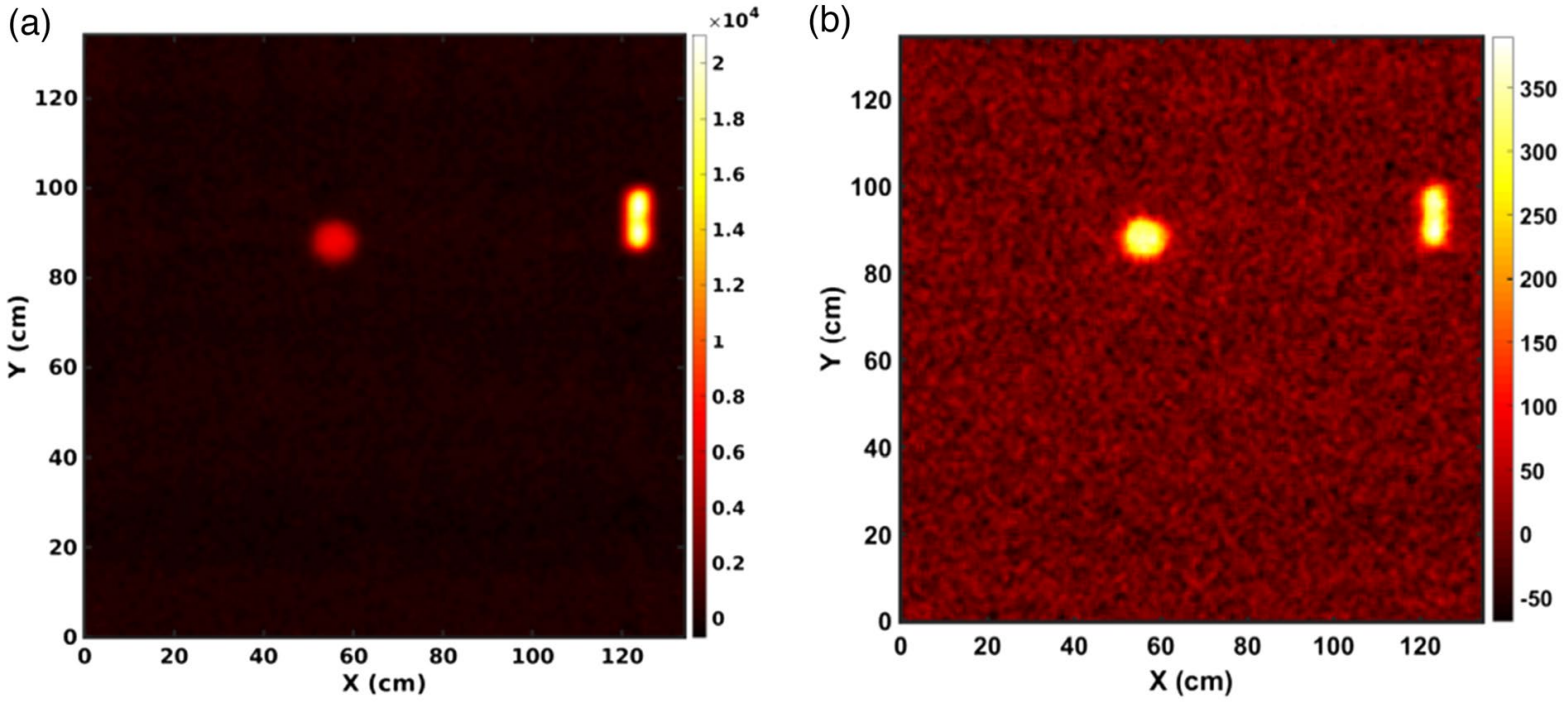
Gamma-ray images of the BeRP ball and Thor core for a 1 h measurement time collected with the CdZnTe-Time encoded imaging system. (**a**) Shows an image with an energy gate of 56–62 keV to focus on the Am-241 line while (**b**) gates on 80–150 keV. In both images, the BeRP ball is placed to the left of the Thor core.

**Figure 9:**
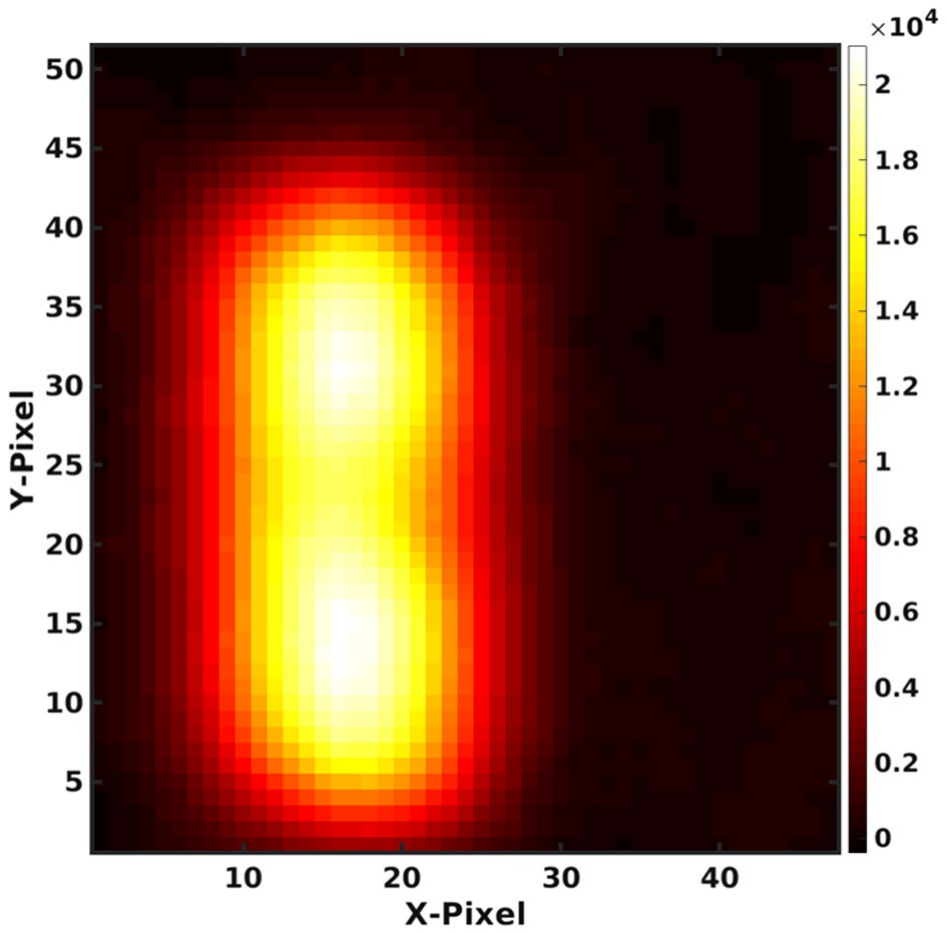
Zoomed image of Fig. 8a showing a close up of the THOR core.

Figure 8 shows the reconstructed images using the CdZnTe time-encoded imaging system. The most significant gamma ray detected in this experiment was the 60 keV emission from americium-241, which is a decay product from plutonium. Photopeaks with energies greater than 60 keV represent isotopes of plutonium, americium, and uranium. Figure 8a shows an image created using only gamma rays from the 60 keV photopeak. The spherical BeRP ball image is a uniform-intensity circle with a good signal-to-noise ratio. The THOR core center section was placed on the edge with its small hole facing the imaging system. Figure 9 shows a zoomed-in version of Fig. 8 and presents a close-up of the THOR core. In this close-up, the slight indentation on the right corresponds to the THOR core hole, which is 1.27 cm in diameter. The resolution could be improved if the mask-to-detector distance were to be increased, which in turn increases the magnification as shown, for example, in Ref.^36^.

Comparing the intensities between the THOR core on the right of the image and the BeRP ball on the left of the image, the THOR core is almost 3 times more intense. This effect is due to the self-shielding differences between the two objects: the outer cladding of the BeRP ball is 12 milli-inches thick steel, compared to only 5 milli-inches thick Ni of the Thor, and the BeRP ball has a higher density than the THOR (19.6 g/cm^3^ versus 16 g/cm^3^). On the other hand, Fig. 8b shows that the intensities are in the same order when using the energy range of 80–150 keV. The difference in image intensity between (a) and (b) relates to the gamma-ray activity of those ranges: the activity of 60 keV emissions from ^241^Am far exceeds the other emissions in the 80–150 keV range.

### Dual-particle imaging

**Figure 10:**
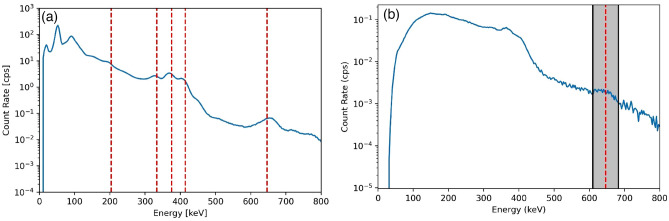
(**a**) Measured gamma ray spectrum of PuO_2_ from the CeBr_3_ singles counts with the following ^239^Pu gamma ray emissions of note: 203 keV, 333 keV, 375 keV, 414 keV, 646 keV. (**b**) Measured gamma-ray coincidence spectrum obtained from stilbene and CeBr_3_ double event interactions. The energy gate used is shown in the highlighted region centered at 646 keV.

**Figure 11:**
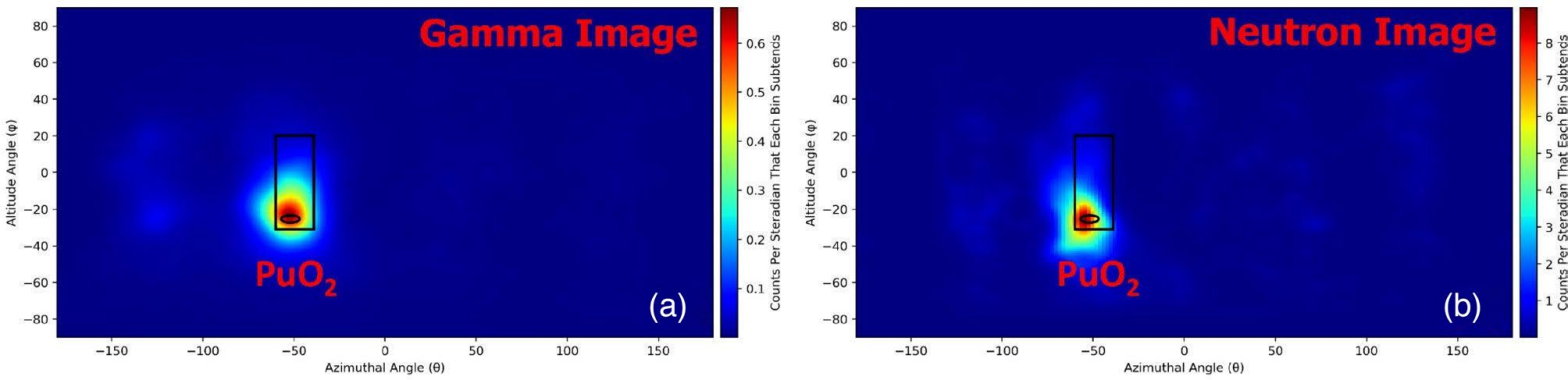
(**a**) Gamma ray and (**b**) neutron images of the NIS6-CAN-1 sintered PuO_2_ source. Note that an energy gate of 646 ± 36 keV was applied for the gamma image to image the 646 keV photopeak. The data was collected from an overnight measurement of 18 hours.

Gamma-ray and neutron imaging and spectroscopy were performed using the H2DPI with a sample of plutonium oxide. Figure 10 shows the gamma-ray energy spectrum measured from the plutonium oxide puck using the H2DPI. ^239^Pu gamma-ray emissions at 203 keV, 333 keV, 375 keV, 393 keV, 414 keV, 640 keV, 642 keV, and 646 keV are all clearly visible in the CeBr_3_ singles spectrum. Gamma-ray Compton imaging was performed using an energy gate around 646 keV, as shown in Fig. 10. Figure 11a shows the resulting gamma-ray image. Energy gating is used during gamma imaging in order to allow for source identification by limiting data to specific energy ranges of interest during the analysis. The 646 keV emission was chosen due to that range not having any other emissions of note during the measurement.

**Figure 12:**
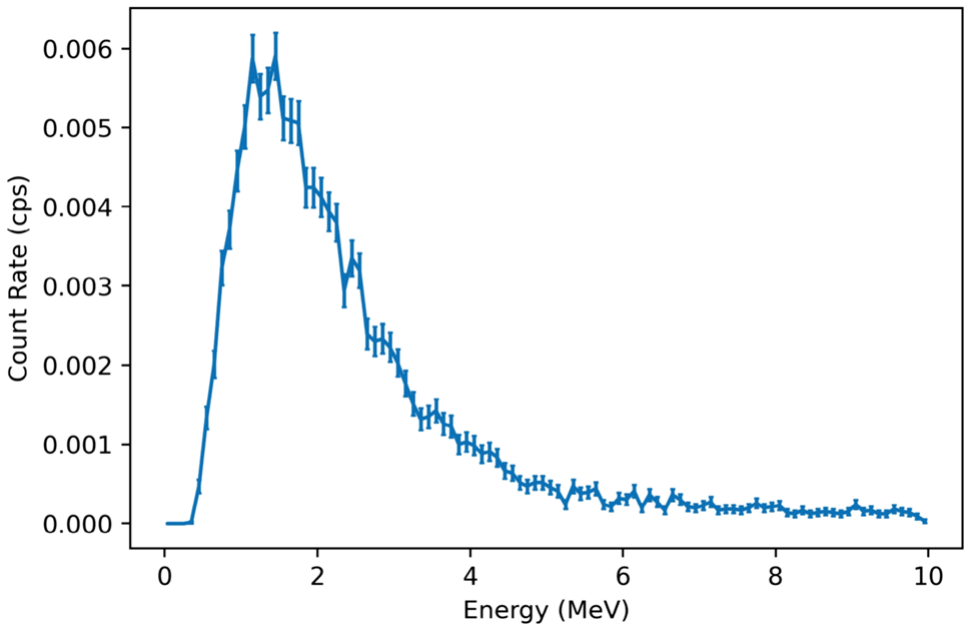
Neutron energy spectrum observed from the H2DPI measurement of PuO_2_. We note that the low-energy cutoff is due to applying a 300 keV lower threshold on individual scatter events.

Neutron imaging was performed on the PuO_2_ to determine if the H2DPI can image neutrons from the contributions of spontaneous fission from ^240^Pu and (α,n) reactions from ^239^ and ^240^Pu on oxygen. The reconstructed neutron image is shown in Fig. 11b. The observed experimental neutron energy spectrum from the PuO_2_ is shown in Fig. 12, and is consistent with the expected spontaneous fission spectrum from ^240^Pu. The low-energy cutoff observed is due to applying a 300 keV energy threshold on individual scatter events, resulting in the drop off below 1 MeV.

In order to converge on the most likely source locations, a converging algorithm, in this case List-Mode Maximum Likelihood Expectation Maximization, is applied for multiple iterations. The black outlines on the images shown in Fig. 11 represent the expected location of the source based on where it was positioned during the measurement. We found that the images obtained using the H2DPI were in agreement with the expected location of the SNM object.

### Active neutron interrogation

**Figure 13:**
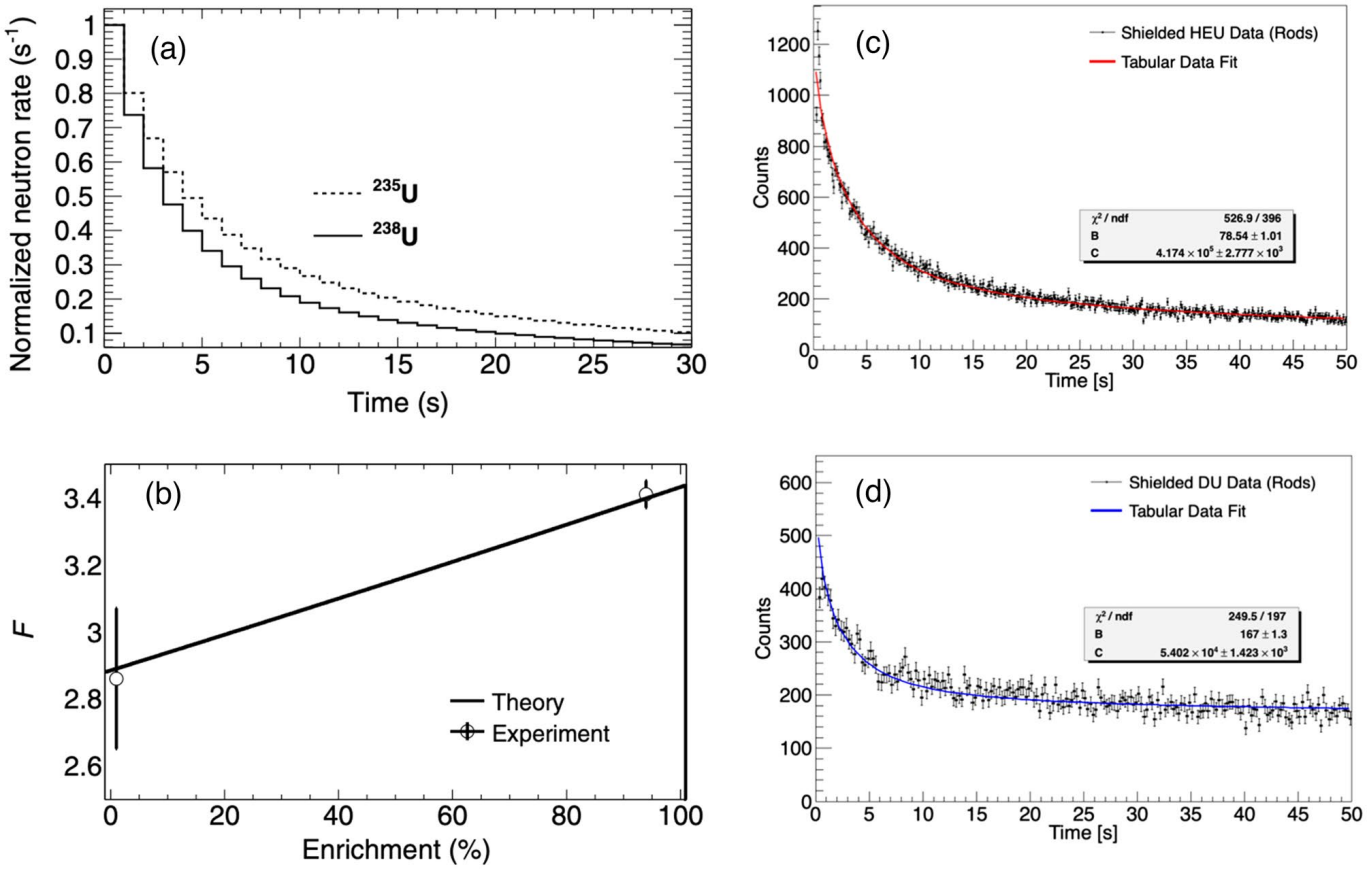
(**a**) Simulated delayed neutron emission profile from ^235^ and ^238^U following active interrogation; (**b**) simulated and measured delayed neutron decay-based discrimination parameter F for ^235^U and ^238^U; (**c**,**d**) HEU and DU delayed neutron emission profiles measured with the rods-type composite scintillator. Corresponding tabular data fits are overlaid. The only fit parameters are the constant background and neutron emission rate at t = 0 (Adapted from^15^ and^17^).

Because the collection of time constants that govern delayed-neutron emission are isotope-specific, the shape of the delayed neutron buildup and decay time profiles are unique to the isotope undergoing fission. As a result, these time profiles can identify and discriminate uranium isotopes. Figure 13a shows the simulated delayed neutron decay time profiles for a 13.8 kg assembly of the Rocky Flats hemishells (^235^U) and a 12.8 kg assembly of similar ^238^U hemishells, illustrating the difference in shape. Experimentally measured decay time profiles for HEU and DU are shown in Fig. 13c,d. The measured profiles showed good agreement with the expected shape calculated based on tabular delayed-neutron group data; the only fitting parameters were a constant background term and the neutron emission rate at time t = 0. Furthermore, a shape parameter, *F*, can be defined for each delayed neutron profile by the ratio *F* = *N*_*1*_*/N*_*2*_, where *N*_*1*_ and* N*_*2*_ are the total number of counts within long (*N*_*1*_*, t*_*0*_ to* t*_*0*_ + 30 s for the decay time profile) and short (*N*_*2*_*, t*_*0*_ to* t*_*0*_ + 3 s for the decay time profile) integration windows^15^. This parameter provides a unique value corresponding to the enrichment of the uranium sample. Figure 13b shows that the *F* values determined from measurements of the HEU and DU test objects agree well with the values predicted by simulation, suggesting that this method may be used to estimate uranium enrichment.

Furthermore, in bulk samples of SNM, delayed neutrons can induce additional prompt fission events in proportion to the fission cross-sections of the isotopes present in the material. These delayed-neutron-induced fission events lead to higher-energy prompt neutrons and coincident radiation in the delayed signal. Because ^235^U has a much higher fission cross-section than ^238^U at typical delayed-neutron energies, the HEU should be distinguishable from DU based on the presence of prompt fission products in the delayed signal. Measurements of the delayed-neutron energy spectrum and the rate of delayed coincidence events confirmed this hypothesis^16^. Finally, delayed neutron profile measurements were conducted with neutron-moderating shielding surrounding the uranium test objects. The results showed that HEU and DU delayed neutron profiles were not significantly distorted by shielding and could still be used as the basis for isotopic discrimination^17^.

## Conclusions

This work demonstrates SNM detection and characterization using gamma-ray and dual particle (fast neutron and gamma ray) imaging and delayed neutron die-away analysis using a DT neutron generator. These experiments were performed using a variety of SNM objects at the NCERC, including uranium and plutonium in both metal and oxide forms.

Gamma-ray imaging was performed with CdZnTe and a principal component analysis for spectral resolution enhancement. This approach demonstrates an improvement from 0.34 to 0.31% FWHM for gamma rays in the 600 keV range. This improved awareness in the energy and spatial domain could aid in verification scenarios or in nuclear safety applications where there might be multiple sources that add background to some desired measurement.

Neutron and gamma-ray dual particle imaging demonstrated the combined imaging and spectrometry to detect, image, and characterize plutonium oxide. We demonstrate imaging of the fast fission neutrons and gamma rays. Gating the gamma-ray imaging on the plutonium emission energies showed that converging on the plutonium oxide location was not only feasible, but can converge accurately with respect to the location of the source. This methodology can also carry over to other sources where specific gamma emissions are of interest. The neutron imaging also converged to the correct source location, which provides an alternative signature if the gamma-ray emissions are obscured by shielding or other intervening material.

In the case of uranium, there is a low rate of spontaneous neutron emission. Active interrogation experiments were performed to induce fission in uranium of varying enrichment. The neutron-interrogated uranium experiments demonstrated that delayed neutron emission time profiles can provide valuable information about the isotopic composition of uranium-containing materials. The profile shapes are derived directly from nuclear data and thus provide a robust means for positively identifying uranium isotopes without needing a calibration standard. We have demonstrated that the delayed neutron profiles for HEU and DU can be accurately measured to close agreement with the shapes predicted from nuclear data. Such measurements can be used to determine the uranium enrichment level.

This work summarized new technologies important in nuclear nonproliferation that were used to measure SNM with masses ranging from 3.3 to 13.8 kg at NCERC. In the future, additional experiments will be performed at NCERC in partnership with the university consortia to further develop technologies for the detection and characterization of SNM.
